# An Evaluation of Sexual Function in the Treatment of Lower Urinary Tract Symptoms Secondary to Benign Prostatic Hyperplasia in Men Treated with the Temporarily Implanted Nitinol Device

**DOI:** 10.1089/end.2022.0226

**Published:** 2022-12-27

**Authors:** Dean Elterman, Mark N. Alshak, Susana Martinez Diaz, Neal Shore, Marc Gittleman, Jay Motola, Sheldon Pike, Craig Hermann, William Terens, Alfred Kohan, Ricardo Gonzalez, Aaron Katz, Jeffrey Schiff, Evan Goldfischer, Ivan Grunberger, Le Tu, Jed Kaminetsky, Bilal Chughtai

**Affiliations:** ^1^ Division of Urology, University Health Network, University of Toronto, Toronto, Canada.; ^2^ Department of Urology, Weill Cornell Medicine, New York, New York, USA.; ^3^ Department of Urology, Carolina Urology Research Center, Myrtle Beach, South Carolina, USA.; ^4^ Department of Urology, South Florida Medical Research, Miami, Florida, USA.; ^5^ Department of Urology, Mt Sinai Hospital, New York, New York, USA.; ^6^ Department of Urology, St John's Episcopal, New York, New York, USA.; ^7^ Department of Urology, Clinical Research Center of Florida, Miami, Florida, USA.; ^8^ Department of Urology, Premier Urology Group, Edison, New Jersey, USA.; ^9^ Department of Urology, Integrated Medical Professionals, Long Island, New York, USA.; ^10^ Department of Urology, Houston Metro Urology, Houston, Texas, USA.; ^11^ Department of Urology, NYU Winthrop Hospital, Long Island, New York, USA.; ^12^ Department of Urology, Premier Medical Group of the Hudson Valley, Poughkeepsie, New York, USA.; ^13^ Department of Urology, New York Methodist Hospital, Brooklyn, New York, USA.; ^14^ Department of Urology, Sherbrooke University Hospital, Sherbrooke, Canada.; ^15^ Department of Urology, Manhattan Medical Research, Manhattan, New York, USA.

**Keywords:** prostatic hyperplasia, lower urinary tract symptoms, minimally invasive surgical procedures, men's health

## Abstract

**Purpose:**

To document the effect of the temporarily implanted nitinol device (iTind; Medi-Tate Ltd, Israel) on sexual function from a multicenter, randomized, single-blinded, sham-controlled trial.

**Materials and Methods:**

Men were randomized 2:1 between iTind and sham procedure arms. The iTind was placed for 5–7 days and an 18F Foley catheter was inserted and removed for the iTind and sham group, respectively. Patients were assessed at baseline, 3, and 12 months postoperatively using the Sexual Health Inventory for Men (SHIM) and International Index of Erectile Function (IIEF). Unblinding occurred at 3 months.

**Results:**

We studied 185 men with a mean age of 61.1 ± 6.5 years. There was no difference in SHIM or total IIEF between iTind and sham at 3 months or in the iTind arm at 12 months compared with baseline. Men in the iTind arm without erectile dysfunction at baseline showed an improvement in total IIEF score of +6.07 ± 21.17 points (*p* = 0.034) at 12 months, in addition to an improvement in ejaculatory function. SHIM scores remained unchanged in all groups, regardless of age, prostate volume, or baseline erectile function.

**Conclusion:**

No changes were observed in sexual and ejaculatory function of patients with iTind regardless of a man's age, prostate volume, and baseline sexual function.

Clinicaltrials.gov: NCT02506465

## Introduction

Benign prostatic hyperplasia (BPH), one of the most common diseases affecting men as they age, often causes lower urinary tract symptoms (LUTS) that can negatively affect daily activities and quality of life (QoL). The impact of BPH is significant, far exceeding other urological diseases, and is forecasted to continue to rise.^1^ Treatments for BPH include pharmacotherapy, and the current gold standard of transurethral resection of the prostate (TURP). However, both are associated with a significant risk to sexual function. Alpha-blockers and 5-alpha reductase inhibitors (5-ARIs) can cause retrograde ejaculation, anejaculation, and reduced libido, leading to 1 year adherence rates as low as 29%.^2^ TURP can also cause ejaculatory dysfunction (EjD) and erectile dysfunction (ED) with rates of retrograde ejaculation of 38.2%–89.0% and ED rates of 13.0%–14.0%.^3^

The sexual side effects associated with both pharmacological and surgical treatment options contribute significantly to the undertreatment of men with bothersome BPH. Since sexual health is a highly important aspect of QoL, especially among younger men or men who are currently sexually active, there is a desire to preserve their sexual function.^2–4^ For this reason, minimally invasive surgical techniques (MISTs) that aim to preserve sexual function have grown in use.^5,6^

There are three novel, U.S. Food and Drug Administration (FDA)-approved MISTs for LUTS secondary to BPH. They include the prostatic urethral lift (UroLift^®^ System; NeoTract-Teleflex, Pleasanton, CA, USA), convective water vapor treatment (Rezum System; Boston Scientific, Marlborough, MA, USA), and the temporarily implanted nitinol device (iTind; Medi-Tak Ltd, Israel). All three treatments have been studied in randomized control trials and have shown to improve LUTS secondary to BPH with no general *de novo* sexual dysfunction relative to sham.^7–9^ However, a more in-depth analysis of the impact of these treatments on sexual function is lacking. In this study, we report an analysis on the effect of iTind on sexual function from a multicenter randomized, single-blinded, sham-controlled trial.

## Materials and Methods

### Study protocol and objectives

Details surrounding this study design have previously been reported.^9^ Briefly, 16 centers (14 in the United States, 2 in Canada) between July 2015 and October 2018 participated in this prospective, randomized, single-blinded study of the second-generation iTind procedure in men with symptomatic BPH. The study was approved by both the FDA and Health Canada, along with Institutional Review Boards at each of the centers. Participants provided written informed consent.

Subjects enrolled included: men ≥50 years, IPSS (International Prostate Symptoms Score) of ≥10, peak urinary flow rate of ≤12 mL/s with a 125 mL voided volume, prostate volume between 25 and 75 cc, and normal urinalysis, complete blood count, and biochemistry. Excluded patients had a postvoid residual volume (PVR) >250 mL, obstructive median lobe (OML) on ultrasound, prostate-specific antigen (PSA) >10 ng/mL or free PSA <25% without negative prostate biopsy, prostate or bladder cancer, previous prostate surgery, neurogenic bladder and/or sphincter abnormalities, recent hematuria or cystolithiasis, current urinary tract infection, decreased renal function, severe respiratory disorders, cardiac disease, immunosuppression, current antithrombotic and antiplatelet treatment, and uncontrolled diabetes mellitus. Cystoscopy was not mandatory during screening, but cystoscopy was used during placement of the device and an intraoperative exclusion criterion of OML existed.

Baseline medical history, BPH-related medications, uroflowmetry, IPSS, PVR, and completion of questionnaires regarding QoL, ED, and EjD was collected at baseline, 6 weeks, 3 months, and 12 months postoperatively. All patients on BPH-related medications started a wash-out period before implantation: 1 month for alpha-blockers and 6 months for 5-ARIs. Medication-naive patients seeking treatment refused medication in preference for a MIST. Patients were unblinded at 3 months.

### iTind procedure

As previously described, the iTind device comprises three elongated, intertwined nitinol struts at the 12, 5, and 7 o'clock positions, an antimigration anchoring leaflet at 6 o'clock, and a polyester retrieval suture for easy device removal.^9^

The device is implanted for 5–7 days, during which it expands and exerts radial force, creating deep ischemic incisions and a remodeling on the prostate tissue at the bladder neck and anterior prostatic fossa. The iTind is deployed under direct visualization in an ambulatory procedure using a rigid cystoscopy. The device is removed through either a rigid cystoscope or an open-ended 22F Foley catheter with topical anesthesia. Both implantation and removal can be done under local, intravenous, or general anesthesia at the discretion of the performing physician. Catheterization is not required following either implantation or removal.

### Sham procedure

The sham control was the insertion and removal of an 18F silicon Foley catheter to simulate both the implantation and retrieval procedures. Throughout the procedure, the surgeon gave verbal description as if deploying the iTind device, after which the catheter was removed. A similar protocol was followed for the sham device retrieval. Although the iTind device is deployed through a rigid cystoscope, a Foley catheter was used to minimize the risk of procedure-related morbidity as suggested by the FDA. Subjects in both the device and control groups were draped to prevent them from seeing the treating physician and the device.

### Questionnaires

We evaluated sexual side effect profiles using validated patient-reported outcomes. The Sexual Health Inventory for Men (SHIM) is a validated tool for screening and diagnosis of ED and severity of ED in clinical practice and research.^10^ SHIM scores are broken down as follows: <5 [no function], 5–7 [severe ED], 8–11 [moderate ED], 12–16 [mild/moderate ED], 17–21 [mild ED], and >21 [no ED].^10^ The SHIM is the same set of questions in the International Index of Erectile Function (IIEF)-5 (abridged form of IIEF-15).

The total version of the IIEF-15 is a validated self-report questionnaire for measuring ED in clinical trials and is widely used. The minimally clinically important difference, the smallest difference in a score that a patient would perceive as beneficial, has been defined as a change of score of 4 points.^11^ In particular, we used question 9 of the IIEF (“When you had sexual stimulation or intercourse, how often did you ejaculate?”) to evaluate ejaculatory function. All questionnaires were completed in the urologist's office at the time of study visit.

### Statistical methodology

Subjects were randomized in 2:1 ratio to either iTind or control (sham) group, respectively. Randomization was conducted using permuted blocks stratified by center by using a central electronic data program.

Subjects were divided into subgroups according to age (three subgroups: 50–60, 61–70, >70), prostate volume (three subgroups: ≤40, 41–60, >60), and baseline total SHIM score (five subgroups: no function, severe ED, moderate, mild/moderate, mild, and no ED). Within each subgroup, change from baseline in total IIEF score was compared between iTind and sham at 3 months visit, and within the iTind group at 12 months visit.

Differences between the groups at 3 months were analyzed using a mixed linear model with group, baseline total IIEF score, and subgroup as predictors. Differences within the iTind group at 12 months were analyzed using a mixed linear model with baseline total IIEF score and subgroup as predictors. Only patients who arrived at 3 or 12 months visits were included in the analysis. Additionally, any patients that required BPH-related medications during the follow-up period were excluded from the analysis.

Statistical analysis was done using SAS 9.4 (SAS Institute, Inc., Cary, NC, USA). Statistical significance was accepted at *p*-value <0.05.

## Results

### Study population

We studied 185 men with a mean age of 61.1 ± 6.5 years who were randomized 2:1 to either iTind (*n* = 128) or sham procedure (*n* = 57). Baseline demographics for the randomized groups were similar, apart from the Charlson Comorbidity Index, which was higher for the iTind group compared with sham (2.52 *vs* 1.26, *p* < 0.001) (Table 1).

**Table 1.** Baseline Demographics

Characteristic	iTIND			Sham			*p*
	*N*	Mean	SD	*N*	Mean	SD	
Age (years)	128	61.5	6.5	57	60.1	6.3	0.1284
BMI	128	28.8	5.7	57	28.8	5.5	1
Height (ft)	128	5.7	0.35	57	5.8	0.32	0.0672
Weight (lbs)	128	194	41.2	57	198	42.7	0.5473
CCI	128	2.52	1.6	57	1.26	0.7	<0.0001
Prostate Volume (mL)	127	43.4	15.5	56	43.8	13.3	0.867
IPSS	127	22.1	6.8	57	22.8	6.2	0.5081
Qmax (mL/s)	125	8.7	3.3	56	8.5	2.4	0.6841
PVR (mL)	125	61.6	55.5	56	61.9	54.2	0.9730
PSA	126	2.2	2.3	56	1.8	1.8	0.2503
QoL	127	4.6	1.3	57	4.9	1	0.1234
IIEF	125	38.3	20.7	57	39.1	19.6	0.8061
SHIM	127	13.2	7.3	57	14.2	6.6	0.3776

Baseline demographics are presented for both iTIND and sham groups.

BMI = body mass index; IIEF = International Index of Erectile Function; IPSS = International Prostate Symptom Score; iTIND = temporarily implanted nitinol device; PSA = prostate-specific antigen; PVR = postvoid residual volume; QoL = quality of life; SD = standard deviation; SHIM = Sexual Health Inventory for Men.

### Sexual health inventory for men

There were no differences in the total SHIM score between iTind and sham at 3 months (13.13 ± 7.88 *vs* 13.00 ± 7.72, *p* = 0.502) and between iTind at baseline and at 12 months (13.98 ± 7.43 *vs* 13.31 ± 7.34, *p* = 0.882). When looking at changes in SHIM at various age groups, there were no differences between iTind and sham at 3 months or iTind at baseline and at 12 months in any age group (50–60, 61–70, and >70 years). Stratifying by prostate volume also did not show any differences between iTind and sham at 3 months or iTind at baseline and at 12 months in <40, 41–60, or >60 mL groups. Lastly, when looking at baseline SHIM scores by severity, there were no differences between iTind and sham at 3 months or iTind at baseline and at 12 months.

### International Index of Erectile Function

There were no differences in the total IIEF score between iTind and sham at 3 months (41.47 ± 22.56 *vs* 39.31 ± 22.43, *p* = 0.645) and between iTind at baseline and at 12 months (42.69 ± 20.52 *vs* 39.24 ± 21.25, *p* = 0.112). There were no differences between iTind and sham at 3 months in any age group or in the iTind group between baseline and 12 months in the older age groups (61–70 and >70 years).

When stratifying by prostate volume, men with smaller prostates (<40 mL), within the iTind arm at 12 months had a change in total IIEF of +4.95 (clinically meaningful), although it was not significant (*p* = 0.08). There was also no change in the total IIEF between iTind and sham at 3 months. At larger prostate volumes (41–60 mL, >60 mL), there continues to be no difference in total IIEF between iTind and sham at 3 months and iTind at baseline and 12 months follow-up.

**FIG. 1.**
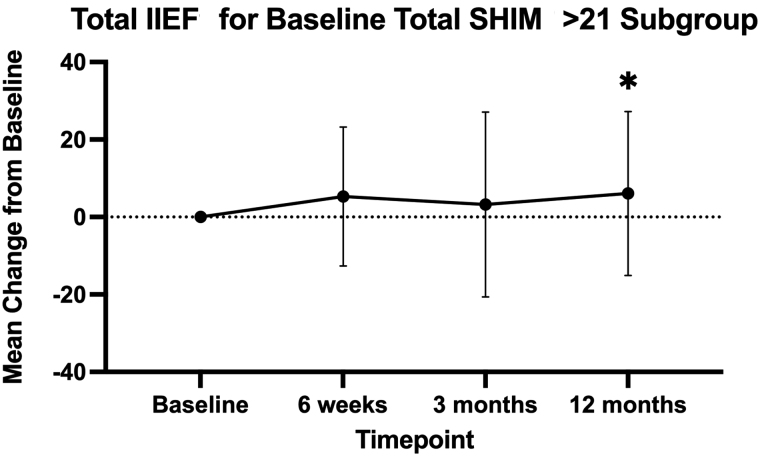
Changes in total IIEF scores from baseline within the iTind subgroup, baseline total SHIM >21. *Statistically significant value (*p* < 0.05). IIEF = International Index of Erectile Function; iTIND = temporarily implanted nitinol device; SHIM = Sexual Health Inventory for Men.

Stratifying by baseline SHIM scores, men treated with iTind who had no ED at baseline showed clinically and statistically significant improvement at 12 months of follow-up in total IIEF score of +6.07 ± 21.17 (*p* = 0.034) (Fig. 1). Men with other levels of SHIM scores did not show any changes either between iTind and sham at 3 months, or from baseline to 12 months follow-up.

### Ejaculatory dysfunction

**FIG. 2.**
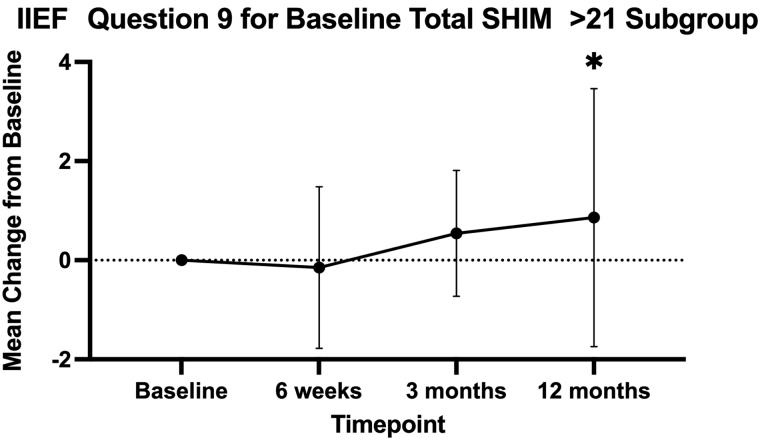
Changes IIEF question 9 (ejaculation upon orgasm) scores from baseline within the iTind subgroup, baseline total SHIM >21. *Statistically significant value (*p* < 0.05).

There were no differences in IIEF question 9 between iTind and sham at 3 months (+0.09 *vs* +0.24, *p* = 0.995) and between iTind at baseline and at 12 months (+0.10, *p* = 0.593). There were no differences when stratifying by age or prostate volume with regard to changes in IIEF question 9 of iTind compared with sham at 3 months or iTind compared with baseline at 12 months. There was an improvement in IIEF question 9 in men without prior ED (SHIM >21) with an average increase of 21.5% (+0.86, *p* = 0.012) (Fig. 2). Men with iTind at other varying levels of baseline ED did not have any differences in IIEF question 9 compared with sham or compared with baseline. Notably, we had no reports of anejaculation, decreased ejaculatory volume, painful ejaculation, or hematospermia.

## Discussion

iTind was an effective treatment for LUTS secondary to BPH while preserving sexual function regardless of age, prostate volume, or baseline ED status. ED, as measured by both total SHIM and IIEF scores and when stratifying by age, prostate volume, or baseline ED status, did not change significantly over the 12-month study period. This is in line with 6-month data from a prospective study showing no ED in men treated with iTind.^12^ Because of the desire to preserve sexual function during the treatment of LUTS secondary to BPH, multiple MISTs have been developed in recent years that aim to treat LUTS while preserving sexual function. Among these MISTs, UroLift and Rezum have both been shown to not cause ED after long-term follow-up.^3^ UroLift showed durability in Male Sexual Health Questionnaire (MSHQ) and IIEF scores when compared with baseline at annual follow-ups for 5 years postimplantation.^7^ Likewise, Rezum showed durability in IIEF and MSHQ scores for 2 years but showed deterioration past this point at 3- and 4-year follow-up points where scores began to drop significantly.^8^

Moreover, iTind was shown to not cause EjD in men, regardless of age, prostate volume, or baseline ED status. Additionally, men without baseline ED had improvement of 21.5% when looking at IIEF question 9 to evaluate for EjD. Importantly, we had no patient-reported ejaculatory adverse events such as anejaculation, decreased ejaculatory volume, painful ejaculation, or hematospermia. Rezum, on the other hand, reported 2.9% anejaculation compared with 0% in their sham arm.^13^ However, this was resolved by 3 months follow-up. Additional studies have estimated the rate of EjD with Rezum to be 3%–6% of men.^3^ Similar to iTind, UroLift has been demonstrated to have no incidence of *de novo* EjD.^14^ Our results are in line with 6-month data, which show ejaculatory function was not only preserved in all 70 cases but also improved according to the Men's Sexual Health Questionnaire-Ejaculatory Dysfunction questionnaire.^12^

Additionally, men who have no baseline ED (SHIM >21) also showed clinically significant improvement of IIEF at 12 months as compared with their baseline (6.07 ± 21.17, *p* = 0.034). It has been demonstrated that there may be a link between LUTS and ED. In a landmark study, men without ED treated with tadalafil showed similar improvement to their LUTS as men with ED, providing evidence that treating LUTS can help improve sexual function.^15^ A recent meta-analysis for UroLift demonstrated that there were significant improvement in both erectile and ejaculatory function at 12 and 24 months following treatment but this effect was less significant at 36 or 48 months follow-up.^16^ Likewise, Rezum was shown to have a 31% improvement in ejaculatory bother score and 27% achieved a minimally clinically important difference in erection function at 1 year follow-up.^17^

Our study has several limitations. First, we experienced loss of follow-up between the baseline groups at 3 months in 29% of iTind patients and 30% of the sham arm. Even though we experienced loss to follow-up, a matched dropout rate between the iTind and sham arm indicates it is likely not a procedure-related drop off. Another important limitation to note is that this study was originally designed to evaluate the change of LUTS following iTind *vs* sham.^9^ As a result, the present findings may be underpowered. Moreover, our evaluation of ejaculatory function was limited to the use of question 9 in IIEF, which may not be the most representative. Nevertheless, the present findings provide the foundation for future work investigating the impact of iTind on sexual function.

## Conclusions

iTind can maintain sexual and ejaculatory function regardless of a man's age, prostate volume, and baseline sexual function.

## Abbreviations Used

5-ARI: 5-alpha-reductase inhibitor
BMI: body mass index
BPH: benign prostatic hyperplasia
CCI: Charlson Comorbidity Index
ED: erectile dysfunction
EjD: ejaculatory dysfunction
FDA: U.S. Food and Drug Administration
IIEF: International Index of Erectile Function
IPSS: International Prostate Symptoms Score
iTind: temporarily implanted nitinol device
LUTS: lower urinary tract symptoms
MIST: minimally invasive surgical technique
MSHQ: Male Sexual Health Questionnaire
OML: obstructive median lobe
PFR: peak urinary flow rate
PSA: prostate-specific antigen
PVR: postvoid residual volume
Qmax: maximum flow rate
QoL: quality of life
SD: standard deviation
SHIM: Sexual Health Inventory for Men
TURP: transurethral resection of the prostate
